# Filarial granuloma of the testicular tunic mimicking a testicular neoplasm: a case report

**DOI:** 10.1186/1752-1947-2-321

**Published:** 2008-10-01

**Authors:** Savio G Barreto, Jude Rodrigues, Roque GW Pinto

**Affiliations:** 1 Department of Surgery, Goa Medical College, Bambolim, Goa, 403 202, India; 2 Department of Pathology, Goa Medical College, Bambolim, Goa, 403 202, India; 3 Department of General and Digestive Surgery, Flinders Medical Center, Adelaide 5046, Australia

## Abstract

**Introduction:**

Any firm or solid intratesticular mass on examination and/or any hypoechoic area within the tunica albuginea on imaging is markedly suspicious for testicular cancer. Filarial involvement of the testicular tunic has not been reported previously.

**Case presentation:**

A 32-year-old man presented with a history of noticing a swelling in his right testicle for a period of 1 month which had become painful over the 10 days before presentation. Pre-operative imaging failed to shed light on the nature of the lesions (malignant or benign). The diagnostic dilemma was explained to the patient and informed consent was obtained for an orchiectomy. The patient underwent a high inguinal orchiectomy. The histopathology revealed a filarial granuloma of the testicular tunic.

**Conclusion:**

While it is generally regarded that any testicular swelling, especially in a young person, should be treated as a malignancy unless proven otherwise, it is important to remember that infectious diseases such as filariasis and tuberculosis may mimic neoplasms. Careful consideration of these diagnoses must be given when dealing with testicular swellings especially in areas where the prevalence of these diseases is high.

## Introduction

Lymphatic filariasis is a major health problem in India with a large number of patients tending to be asymptomatic. Genital filariasis in India more commonly presents as a secondary vaginal hydrocele with an associated epididymo-orchitis. While testicular involvement is rare, the discovery of an adult worm in the testicular tunics has not been reported previously. We thus outline this unique presentation of testicular filariasis.

## Case presentation

A 32-year-old man presented with a history of noticing a swelling in his right testicle over a period of 1 month but which had become painful over the 10-day period before presentation. The patient was afebrile and had not suffered any trauma to the testes. Examination of the genitalia revealed a mild enlargement of the right testicle with a palpable ill-defined tender 1 cm × 1 cm nodule at the lower pole of the right testis. The epididymis and spermatic cord were normal to palpation. The left testis and cord were normal. Complete blood count, including the differential count, was normal (no eosinophilia). His chest X-ray was normal. Tests for serum tumour markers were not performed as the patient could not afford these. A scrotal ultrasound demonstrated the right testicle to be larger than the left. There was evidence of a 1.5 cm × 0.6 cm hypoechoic lesion in the region of the right testicular lower pole parenchyma with an echogenic speck within it, suggestive of a testicular neoplasm. The patient was counselled on the likelihood of this being a benign tumour and that we could not rule out a malignancy with entire certainty based on the information available to us. Since the swelling was painful, the patient consented to an orchiectomy. The patient underwent a right inguinal orchiectomy. Intraoperative findings included an enlarged testicle with a 1.5 cm × 0.5 cm hard nodule within the lower pole of the testis. The nodule was away from the epididymis. The epididymis and cord were normal. There was no associated hydrocele. The histopathology revealed, "a granuloma comprising of central adult filarial worm surrounded by epithelioid cells, lymphocytes and fibroblasts in the tunica of the lower pole. No significant pathology was detectable in the rest of the testis, epididymis or the spermatic cord". In retrospect, the patient was subjected to three midnight blood smear examinations and a buffy coat smear examination, which revealed no evidence of filarial infection.

## Discussion

**Figure 1:**
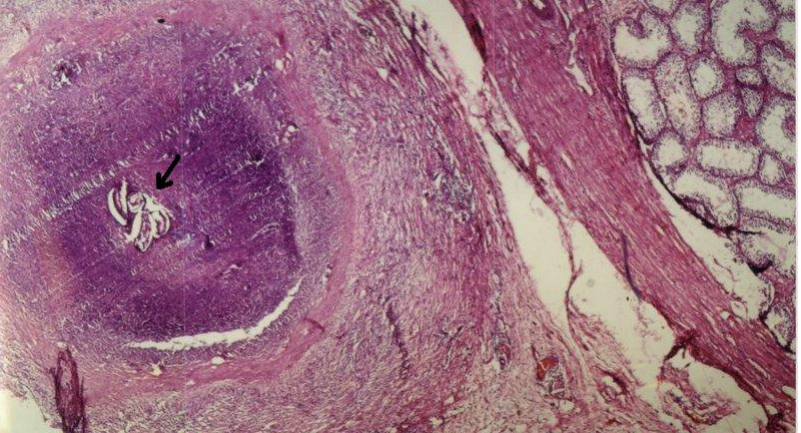
**Adult filarial worm in the testicular tunic**. Histopathologic section showing the adult filarial worm in the paratesticular region surrounded by a granuloma. In the top right corner, the normal seminiferous tubules are seen (haematoxylin & eosin, ×40).

While filarial orchitis is a rare, yet reported manifestation [1,2], to the best of our knowledge based on a thorough PubMed search (keywords: *filarial, worm, tunica, albuginea, vaginalis*), this is probably the first case of a filarial granuloma occurring in the tunica of the testis (Fig. 1).

Filarial lesions of the paratestis occur in the stage of early established filarial infection [2]. Secondary to the death of the worm, a filarial granuloma is said to form consisting of surrounding blood vessels and the perilymphatic tissue that contain an abundance of eosinophilic cells. This, together with an aggregation of epithelioid cells and foreign body giant cells around the dead worm, results in the granuloma. Clinically, it presents as a tender, well-circumscribed, firm nodule in relation to the epididymis.

Tissue eosinophilia is a useful diagnostic hint [2], though it may be absent as seen in our case. The role of Fine Needle Aspiration Cytology (FNAC) in the diagnosis of testicular and epididymal lesions is controversial. While FNAC has been used in countries such as India where the prevalence of genital tuberculosis and filariasis is high [3], this is not advisable considering the possibility of disease dissemination should the lesion be malignant. Ultrasonography remains the imaging method of choice for any intrascrotal pathology. However, the varied appearances of benign lesions often preclude a clear differentiation of benign from malignant neoplasms. Calcified dead worms [4] following diethylcarbamazine (DEC) treatment presenting as specks of calcification seen on imaging and the classic sign for filariasis, the 'filarial dance sign', caused by the undulating movements of the live adult worms [5], can help diagnose filarial involvement of the testes and scrotum with a reasonable degree of certainty. However, as seen in our case, reaching a pre-operative diagnosis of testicular filariasis can be difficult. The use of nuclear magnetic resonance spectroscopy has been studied in vitro and in animal models using *Brugia malayi *[6]. This investigation is as yet experimental.

The diverse neoplastic and non-neoplastic lesions that occur in the paratesticular region include neoplasms of mesenchymal origin, viz. malignant mesothelioma, adenomatoid tumours, abnormalities of testicular appendages, non-neoplastic cystic lesions, viz. mesothelioma cyst and reactive mesothelioma hyperplasia, malakoplakia, sarcoidosis and inflammatory pseudotumour. Also important in the differential diagnosis would be tuberculosis [1].

Diethylcarbamazine (DEC) is considered the drug of choice. Since the disease simulates clinical malignancy, it is often the cause of unilateral orchiectomy [2]. While criteria for surgical intervention have not yet been formulated [2], testicular-sparing surgery could probably present a useful modality in the management algorithm of such patients [7].

## Conclusion

While it is generally regarded that any testicular swelling, especially in a young person, should be treated as a malignancy unless proven otherwise, it is important to remember that infectious diseases such as filariasis and tuberculosis may mimic neoplasms. Careful consideration of these diagnoses must be given when dealing with testicular swellings especially in areas where the prevalence of these diseases is high.

## Competing interests

The authors declare that they have no competing interests.

## Authors' contributions

SGB collected the patient data and references and prepared the manuscript. RGWP performed the histological examination of the testis, and reviewed the scientific content of the manuscript. JR was involved in the surgery and was a major contributor in writing the manuscript and its final review. All authors have read and approved the final version of the manuscript.

## Consent

Written informed consent could not be obtained in this case since the patient is lost to follow-up. We believe that this case report contains a worthwhile clinical lesson which could not be made as effectively in any other way. We expect that the patient would not object to the publication since every effort has been made so that he remains anonymous.
